# Perspectives for treating Alzheimer's disease: a review on promising pharmacological substances: Perspectivas no tratamento da doença de Alzheimer: uma revisão sobre substâncias farmacológicas promissoras

**DOI:** 10.1590/1516-3180.2015.01980112

**Published:** 2015-11-13

**Authors:** Maurílio de Souza Cazarim, Julio Cesar Moriguti, Abayomi Tolulope Ogunjimi, Leonardo Régis Leira Pereira

**Affiliations:** 1 MSc. Doctoral Student in the Department of Pharmaceutical Sciences, School of Pharmaceutical Sciences of Ribeirão Preto, Universidade de São Paulo (USP), Ribeirão Preto, SP, Brazil.; 2 MSc, PhD. Associate professor (MS-5) in the Department of Internal Medicine, Ribeirão Preto Medical School, Universidade de São Paulo (USP), Ribeirão Preto, SP, Brazil.; 3 MSc, Professor in the Department of Pharmaceutics, Faculty of Pharmacy, Obafemi Awolowo University, Nigeria. Doctoral Student, School of Pharmaceutical Sciences of Ribeirão Preto Universidade de São Paulo (USP), Ribeirão Preto, SP, Brazil.; 4 MSc, PhD. Professor of the Department of Pharmaceutical Sciences, School of Pharmaceutical Sciences of Ribeirão Preto, Universidade de São Paulo (USP), Ribeirão Preto, SP, Brazil.

**Keywords:** Alzheimer disease, Molecular mechanisms of pharmacological action, Drug therapy, Amyloid beta-peptides, Tauopathies, Doença de Alzheimer, Mecanismos moleculares de ação, farmacológica, Quimioterapia, Peptídeos beta-amiloides, Tauopatias

## Abstract

**CONTEXT AND OBJECTIVE:**

Dementia is a syndrome characterized by functional and cognitive decline. Alzheimer's disease (AD) is one of the most common causes of dementia and has high prevalence among the elderly. It is known that there is no drug capable of interfering with the course of the disease. Research on treatments for AD has been marked by the appearance of new drugs and their abandonment. This study aimed to describe drugs that have been studied with regard to treating AD and which are capable of influencing the course of the disease.

**DESIGN AND SETTING:**

Narrative review on original articles published worldwide.

**METHODS:**

A systematized search was conducted in the PubMed/MEDLINE, Cochrane Library/Cochrane and SciELO/Bireme databases. The descriptors "Molecular Mechanisms of Pharmacological Action" and "Drug Therapy" were each combined with the descriptor "Alzheimer disease". All of these can be found in MeSH and DeCS. These descriptors were used alone or in combination, and a filter specifying publication between January 2009 and October 2015 in English, Spanish or Portuguese was set.

**RESULTS:**

6,888 articles were found, of which 37 were included in this review; 70.3% of the articles selected were of good quality with low or unclear risk of bias. 86 drugs were considered promising for AD treatment and these were classified into 20 pharmacological categories.

**CONCLUSION:**

There are no drugs capable of influencing the course of AD such that treatments are safe and effective. However, immunomodulators stood out as promising, given their effectiveness and quality in the articles analyzed.

## RESUMO

**CONTEXTO E OBJETIVO:**

A demência é uma síndrome caracterizada por declínio funcional e cognitivo, sendo a doença de Alzheimer (DA) uma das causas mais comuns e de alta prevalência em idosos. Sabe-se que não há medicamento capaz de interferir no curso da doença e as pesquisas para o tratamento da DA têm sido marcadas pelo surgimento e abandono de novas drogas. O objetivo deste estudo foi descrever as drogas capazes de influenciar o curso da DA que têm sido estudadas para o tratamento da doença.

**TIPO DE ESTUDO E LOCAL:**

Revisão narrativa de artigos originais publicados mundialmente.

**MÉTODOS:**

Foi realizada uma busca sistematizada nas bases de dados PubMed/MEDLINE, Cochrane Library/Cochrane e SciELO/Bireme. Cada um dos seguintes descritores "Mecanismos Moleculares de Ação Farmacológica" e "Quimioterapia" foram combinados com o descritor "Doença de Alzheimer", todos encontrados no MeSH e DeCS. Os descritores foram usados sozinhos ou em combinação, fixando como filtros as publicações de 2009 a 2015, em língua inglesa, espanhola e portuguesa.

**RESULTADOS:**

Foram encontrados 6.888 artigos, dos quais 37 foram incluídos nesta revisão; 70,3% dos artigos selecionados tiveram boa qualidade com baixo ou indefinido risco de viés. Foram elencadas 86 drogas promissoras ao tratamento da AD. Elas foram classificadas em 20 categorias farmacológicas.

**CONCLUSÃO:**

Não há fármacos capazes de interferir no curso da DA com efetividade e segurança no tratamento. Contudo, os imunomoduladores foram considerados promissores devido ao fato de apresentarem efetividade e qualidade nos artigos analisados.

## INTRODUCTION

Dementia is a syndrome characterized by functional and cognitive decline.^1^^,^^2^^,^^3^^,^^4^ Alzheimer's disease (AD) is one of the several possible causes of dementia, corresponding to 60% to 70% of cases.^4^^,^^5^ The prevalence of dementia due to AD increases with age, such that AD accounts for 5% of dementia cases in the age group of 65-74 years and 50% in the age group over 85 years.^6^ AD is responsible for reduction of life expectancy by 50% from the time of diagnosis in elderly patients.^7^

AD is characterized by destruction of the functional activity of neurons in the cerebral cortex, amygdale, frontal base, limbic system and hippocampus, and also by cortical atrophy, thereby causing impairment of cholinergic synapses in the central nervous system (CNS). This is due to formation of inflammatory plaque or neuritic plaque (NP) and neurofibrillary tangles (NFTs), which are associated with the first onset of disease and secondary development thereafter. The brain regions affected account for memory, learning, emotional reactions and behavior.^8^^,^^9^^,^^10^

The mechanisms for formation of NP and NFTs that have been best elucidated relate to amyloid-beta (Aβ) peptide and tau protein. Some studies have indicated possibilities that represent the "start" of NP and NFT formation, such as inflammation, mitochondrial function, oxidative stress, vascular changes, gene expression and functionality of the endocrine system. These may be factors relating to the physiopathology of AD.^4^^,^^10^

Aβ peptide is a natural product from the metabolism of the amyloid precursor protein (APP), which is a neuronal transmembrane protein.^8^^,^^11^ Aggregation of Aβ in the brain and in the walls of cerebral blood vessels gives rise to extracellular lesions that lead to formation of NP, thus causing neurotoxicity. Overproduction of APP or diminished clearance are possible explanations for the occurrence of this process. These situations arise through mutation of both genes that encode APP (chromosome 21) and in the genes encoding presenilin 1 and output 2 (chromosomes 14 and 1, respectively).^10^^,^^12^^,^^13^

NFT formation can be explained in terms of hyperphosphorylation of tau protein filaments. This protein is important for formation of the neuronal cytoskeleton and for transport through formation of microtubules. Thus, this hyperphosphorylation involves denaturing the protein that takes part in intracellular transport, which culminates in neuronal cell death.^10^^,^^12^

Over almost the entire course of the disease, cholinergic activity is most affected and this is correlated with the severity of AD. The reduction in the number of cholinergic neurons through development of AD implies loss of nicotinic receptors in the hippocampus and cortex. These are responsible not only for release of acetylcholine (ACh), but also for release of other important neurotransmitters that are involved in memory and mood, including glutamate, serotonin and norepinephrine.^10^

Noradrenergic and serotonergic systems are also impaired through loss of neurons in the locus coeruleus and raphe nuclei.^14^ Glutamate receptors, particularly of the type N-methyl-D-aspartate (NMDA), are continuously activated with lower concentrations of glutamate, thereby resulting in stimulation of uncoordinated neurons and hyperarousal mediated by increased calcium influx.^15^ This leads to destruction of neurons, with cortical atrophy, which then leads to ventricular enlargement and impairment of different neurotransmission pathways in key regions responsible for memory, learning, emotional reactions and behavior.^14^^,^^16^

In this context, the guidelines established in pharmacological therapies for treating AD can be summarized as inhibition of degradation of ACh or blocking of glutamate receptors, thereby reducing glutamatergic activity. This has the aim of enhancing cholinergic activity and decreasing the hyperactivity of the excitatory neurotransmitter glutamate in the cortex and hippocampus regions.^10^

Accordingly, the drugs commonly used in current clinical practice are acetylcholinesterase inhibitors (IAChEs): donepezil, galantamine and rivastigmine, which can be used alone or in combination with memantine, an antagonist of NMDA receptors that can also be used alone, depending on the stage of the disease. However, the pharmacological agents so far available as drug therapy have not been proven to modify the course of the disease, since they are only effective for symptomatic treatment.^10^^,^^17^

In fact, success in this quest has consistently required clarification of the molecular mechanisms relating to the two best pathological pathways elucidated with regard to formation of NP and NFTs in AD cases.^18^^,^^19^^,^^20^^,^^21^ Some studies have put forward new biomolecular mechanisms linked to the physiopathology of AD, which could become possible therapeutic targets within the treatment for this disease. Many of these are called alternative targets and are justified because they are closely linked to neuroregeneration, reduction of neurotoxicity and promotion of positive effects on neuronal homeostasis.^18^^,^^21^ However, it has been reported in the literature that only 30% of the compounds studied with a view to treating AD are molecules with mechanisms of action that are directly related to the above pathophysiological pathways, i.e. molecules that are able to modify the course of the disease. Within this percentage, almost 90% do not have a very clear therapeutic target, while about 10% are directed towards alternative targets requiring further elucidation about their association with AD.^18^^,^^21^^,^^22^

According to evidence in the literature, drug treatments for Alzheimer's disease had not presented any innovations up to the year 2015 and there were no drugs that would be able to combat the pathophysiology of AD. In this context, it is necessary to elucidate the ways in which the search for new drugs has always been conducted. In this manner, new proposals for drug therapies that have been studied for treating AD can be disseminated, thus strengthening research on new drugs and providing updates for the scientific community with regard to promising drug treatments.

In addition, the development of new drugs for treating AD has been marked by the appearance of new possibilities and their subsequent abandonment. This difficulty encourages the need to search for new treatments that could influence the course of AD, through a review of the literature, given that many clinical trials have shown that some drugs are likely to be promising, yet these studies were inconclusive.^18^^,^^22^

## OBJECTIVE

The present study had the aim of conducting a review, among publications indexed in scientific databases, of the studies on drug and chemical groups that have been investigated in relation to treating AD over the last five years, highlighting those with promising results in terms of their propensity to modify the course of AD.

## METHODS

This study consisted of a narrative review conducted through a qualitative assessment on the articles analyzed. The search was conducted in the PubMed database (MEDLINE) (http://www.ncbi.nlm.nih.gov/pubmed/), Cochrane Library database (Cochrane Collaboration) (http://www.cochranelibrary.com/) and SciELO database (Bireme) (http://www.scielo.br/). MeSH (Medical Subject Headings) and DeCS (Descriptors in Health Sciences) descriptors were used. The descriptors "Alzheimer disease", "Molecular Mechanisms of Pharmacological Action" and "Drug Therapy" were used alone or in combination, as follows: "Alzheimer disease"; "Alzheimer disease" AND "Molecular Mechanisms of Pharmacological Action" AND "Drug Therapy"; "Alzheimer disease" AND "Molecular Mechanisms of Pharmacological Action"; "Alzheimer disease" AND "Drug Therapy". Studies in the English, Spanish and Portuguese languages were taken into consideration. We set a filter to limit the search to articles published from January 2009 to October 2015.

In addition, the review was divided into two stages. In the first stage, only review articles were selected, including narratives, systematic reviews and systematic reviews with meta-analyses. At this stage, we sought to answer the following question: "What new drugs that have been studied in relation to treating AD would be capable of influencing the course of the disease?" The second stage involved a search for articles that discussed laboratory studies, human studies, observational studies and clinical trials on the drugs or chemical groups found in the first stage. The second search was conducted using the same specifications as in the first search, but the descriptors were modified such that they became "Alzheimer disease" and the name of the drugs or chemical groups.

Articles were identified and all duplicate records were excluded. Initially, the title and abstract were read in order to include original articles in which the main objective was to describe the pharmacotherapy of AD. Therefore, original articles relating to AD that did not include drugs, molecules or substances for pharmacological treatment were excluded. To ascertain the eligibility criteria, we used the manual of the Brazilian Academy of Neurology.^23^ The Cochrane classification criteria for risk of bias from the Cochrane Bias Methods Group were used to assess the quality of the articles selected.^24^ This classification was made by two researchers and, when necessary, a third researcher gave his opinion.

## RESULTS

**Figure 1:**
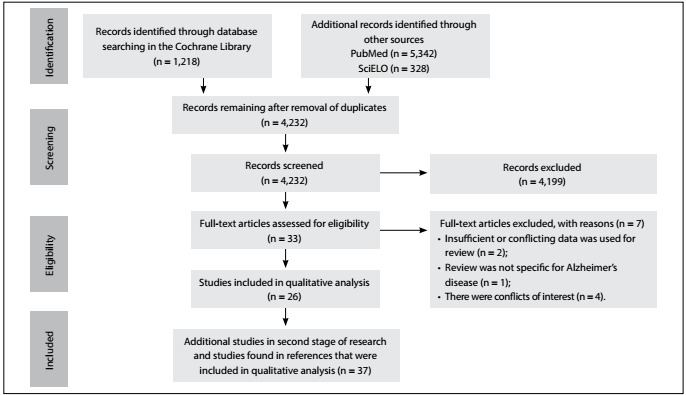
Flowchart of article selection

The search identified 6,888 studies, of which 37 were included in this study (Figure 1). All the articles that were in accordance with our inclusion criteria were available as full texts.

Among the articles selected for this study, the United States were seen to be the most important source country for publications, with 15 articles (40.5%); followed by Germany, with 4 articles (10.8%), and Canada, with 3 articles (8.1%). Regarding the quality of the articles accessed in the first part of this review, 7 (26.5%) were identified as presenting low risk of bias, 12 (46.1%) were identified as presenting unclear risk of bias and 7 (26.5%) were identified as presenting high risk of bias. In all the articles selected for this study, the percentages were 12 (32.5%), 14 (37.8%) and 11 (29.7%), respectively for the three categories of quality (Table 1).

**Table 1:** Results from the articles selected for this study

Reference	Authors, year and country	Therapeutic classes	Results basis	Study design	Cochrane risk of bias classification*
18	Léon et al., 2013 (England)	Cholinesterase inhibitors; statins: lipid-modifying agents; antioxidants; chelating agents	Clinical evidence	Narrative review	Unclear
19	Konrath et al., 2013 (Brazil)	Cholinesterase inhibitors; alkaloids	Not specific	Narrative review	High
20	McGuinness et al., 2013 (Ireland)	Statins: lipid-modifying agents	Phase III clinical trial	Systematic review (randomized with meta-analysis)	Low
21	Rubio-Perez and Morrilas-Ruiz, 2012 (Spain)	Anti-inflammatory agents; cytokines	Not specific	Narrative review	High
22	Appleby et al., 2013 (USA)	Agents for treating diabetes mellitus; nicotinic receptor agonists; phosphodiesterase inhibitors	Clinical evidence	Narrative review	Unclear
25	Pettenati et al., 2003 (Italy)	Cholinesterase inhibitors; immunomodulators; anti-inflammatory agents; NMDA receptor antagonists; antioxidants; phospholipase A2 inhibitors; nootropic drugs without elucidated mechanism of action for Alzheimer's disease; hormone therapy agents; chelating agents	Clinical evidence	Narrative review	Unclear
26	Sun et al., 2012 (China)	Cholinesterase inhibitors; muscarinic agonists; immunomodulators; anti-inflammatory agents; statins: lipid modifying agents; antioxidants; chelating agents; antihypertensive agents	Clinical evidence	Narrative review	Unclear
27	Yoo and Park, 2012 (Korea)	Terpenoids; antioxidants	Not specific	Narrative review	High
28	Dodel, 2013 (Germany)	Immunomodulators	Clinical evidence	Narrative review	Unclear
29	Mikulka et al., 2014 (USA)	Immunomodulators; secretase inhibitors; Inhibitors or modulators of beta and gamma-secretase	Phases I, II and III clinical trial	Systematic review (with clinical trial)	Low
30	Shukla et al., 2012 (USA)	Immunomodulators; cyclin kinase-5 dependent modulators	Not specific	Narrative review	High
31	Doody et al., 2014 (Germany)	Immunomodulators	Phase III clinical trial	Randomized clinical trial (double blind)	Low
32	Salloway et al., 2014 (USA)	Immunomodulators	Phase III clinical trial	Randomized clinical trial (double blind)	Low
33	Enciu and Popescu, 2013 (Romania)	Anti-inflammatory agents	Clinical evidence	Narrative review	Unclear
34	Feng and Wang, 2012 (China)	Anti-inflammatory agents; antioxidants; phospholipase A2 inhibitors	Not specific	Systematic review	High risk of bias
35	Wolfe, 2012 (USA)	Anti-inflammatory agents; Inhibitors or modulators of beta and gamma-secretase; chelating agents	Clinical evidence	Narrative review	Unclear
36	Ghosh et al., 2012 (USA)	Inhibitors or modulators of beta and gamma-secretase	Phase II clinical trial	Narrative review	Low
37	Hopkins, 2011 (USA)	Inhibitors or modulators of beta and gamma-secretase	Phase III Clinical trial	Short report	Unclear
38	Piccinni et al., 2013 (Italy)	Inhibitors or modulators of beta and gamma-secretase	Not specific	Narrative review	High
39	Eschecerria et al., 2012 (USA)	Nicotinic receptor agonists	Clinical evidence	Narrative review	Unclear
40	Freiherr et al., 2013 (Germany)	Agents for treating diabetes mellitus	Clinical evidence	Narrative review	Unclear
41	Morris and Burns, 2012 (USA)	Agents for treating diabetes mellitus	Clinical evidence	Narrative review	Unclear
42	Xu et al., 2015 (China)	Agents for treating diabetes mellitus	*In vitro*, in laboratory	Experimental study	High
43	Papadopoulos et al., 2013 (Canada)	Agents for treating diabetes mellitus	*In vivo*, in laboratory	Experimental study	High
44	Fukasawa et al., 2012 (Japan)	Retinoids	*In vivo*, in laboratory	Experimental study	High
45	Wong et al., 2013 (USA)	Statins: lipid-modifying agents	Not specific	Systematic review (randomized with meta-analysis)	Unclear
46	Calcul et al., 2012 (USA)	Polyphenols	Clinical evidence	Narrative review	Unclear
47	García-Osta et al., 2012 (Spain)	Phosphodiesterase inhibitors	Clinical evidence	Narrative review	Unclear
48	Evans et al., 2014 (USA)	Antioxidants; NMDA receptor antagonists	Phase III clinical trial	Randomized controlled clinical trial	Low
49	Revett et al., 2013 (Canada)	Antioxidants; NMDA receptor antagonists	Not specific	Narrative review	High
50	Kellermann et al., 2011 (Germany)	Antioxidants	Phase III clinical trial	Systematic review (randomized with meta-analysis)	Low
51	Freund Levi et al., 2014 (USA)	Antioxidants	Phase II clinical trial	Randomized controlled clinical trial	Low
52	Sun et al., 2012 (USA)	Phospholipase A2 inhibitors	Not specific	Narrative review	High
53	Koliaki et al., 2012 (Greece)	Nootropic drugs without elucidated mechanism of action for Alzheimer's disease	Phase III clinical trial	Randomized controlled clinical trial	Low
54	Maki and Henderson, 2012 (USA)	Hormone therapy agents	Randomized clinical trial	Narrative review	Low
55	Anekonda and Quinn, 2011 (Canada)	Antihypertensive agents	Clinical trials	Narrative review	Low
56	Valenzuela et al., 2012 (Australia)	Antihypertensive agents	Randomized controlled clinical trial	Narrative review	Low

*We used the classification criteria of the Cochrane risk of bias to access the quality of the articles. In the case of review articles, the results from the selected articles were accessed in order to ascertain the evidence that was present in the kind of study that generated the results. Consequently, the Cochrane classification was made based on this evidence.

In an attempt to gain better insight into the pharmacological substances found in this review, they were classified using chemical groups as described by the ATC/DDD of the World Health Organization, whenever possible. These groups were classified based on their action on therapeutic targets relating to the course of the disease. There were groups under investigation that effectively act on the course of the disease, on targets relating to physiopathology that have not been well elucidated, and groups with therapeutic targets not clarified for the disease course. There was another group that acts on alternative therapeutic targets that have an association with the newly discovered pathophysiological pathways of AD; this group was classified as an alternative therapeutic target for the course of the disease. There was also the group of substances in which the mechanism of action of the drug is not related to any specific therapeutic target, but associated with pathophysiological mechanisms already accounting for AD; this group has been classified as a group of potential modifiers of the course of the disease without specific therapeutic target (Table 2).

**Table 2:**
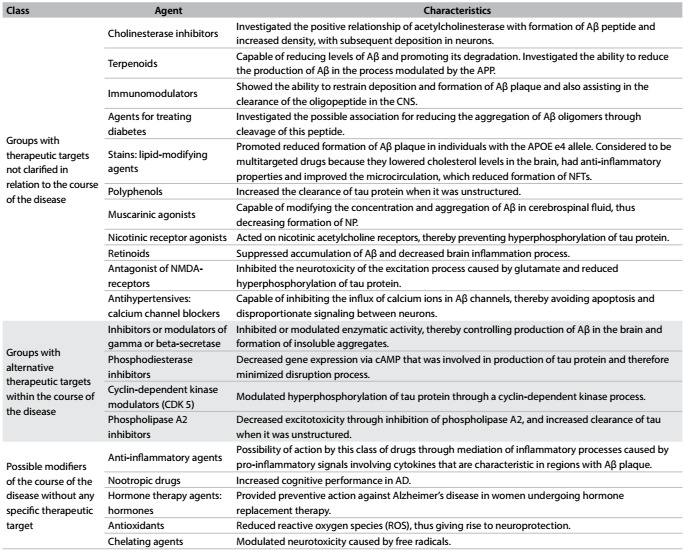
Classification of pharmacological groups that showed the prospect of changing the course of the disease, with regard to action on therapeutic targets

NP = neuritic plaque; NFT = neurofibrillary tangles; CNS = central nervous system; APP = amyloid precursor protein; Aβ = amyloid-beta; APOE = apolipoprotein E; NMDA = N-methyl-D-aspartate; cAMP = cyclic adenosine monophosphate; CDK5 = cyclin-dependent kinase 5.

## DISCUSSION

### Cholinesterase inhibitors

#### Alkaloids: huperzine, aporphyrin, lycorine and quaternary beta-carboline

Although this is a chemical group used for symptomatic treatment of AD in the clinical phase of the disease, its possible mechanism of action with regard to changing the course of the disease has been investigated. This source is based on the positive relationship of the enzyme cholinesterase (ChE) with formation of Aβ oligomers and increased density, with subsequent deposition in neurons.^25^

Cholinesterase inhibitors (ICHE) appear to be promising drugs for treating AD, based on the action of some compounds of this enzyme. Inhibiting alkaloids such as physostigmine and galantamine are used in conventional treatment. Thus, some natural alkaloids have been investigated in an attempt to highlight changes to the course of AD. Among these compounds are the steroidal alkaloid triterpene, which promotes non-competitive inhibition; quinolizidine and lycopodium alkaloids such as huperzine, which promotes competitive inhibition; isoquinolines such as aporphyrin and benzylisoquinoline, which are non-competitive compounds, and galantamine and lycorine, which are competitive; and indole alkaloids, which are divided into indole alkaloids, monoterpenes and physostigmine, which are competitive, and quaternary beta-carbolines, which are non-competitive.^18^^,^^19^^,^^25^

Out of all of these, we highlight huperzine as a potent, selective and reversible inhibitor of AChE. This is used in cases of myasthenia gravis, organophosphate poisoning and schizophrenia. Clinical trials on huperzine have shown that its use improves short-term memory, but there is need for more robust studies in order to prove its effectiveness and minimal toxic effects, which are due to its high selectivity and its ability to cross the blood-brain barrier (BBB).^19^^,^^26^

### Muscarinic agonists

#### Xanomeline and milameline

These substances are capable of modifying the CSF concentration of Aβ aggregation and decreasing formation of amyloid plaque.^25^ Xanomeline and milameline have the ability to cross the BBB and have important effects relating to cognitive improvement, but they also have significant side effects on the cardiovascular and gastrointestinal tracts.^26^

### Terpenoids

#### Ginseng

Ginsenosides are natural compounds extracted from the plant *Panax ginseng* . They have been the subject of *in vitro* clinical trials aimed at reducing the levels of Aβ and promoting its degradation. They have the ability to suppress production of Aβ, in a process modulated by APP. The results from animal studies have shown that ginsenosides are effective in relation to attenuating some neuroinflammation markers, improving spatial perception and increasing synaptic density.^27^

### Immunomodulators

#### Gammagard Liquid, bapineuzumab, solanezumab, crenezumab, gantenerumab and Affitope

Passive immunization has been shown to reduce the brain levels of Aβ oligomers, increase their clearance and redistribute them through the circulatory system, to other brain areas and tissues that favor their elimination.^25^ One immunoglobulin with important effects in relation to AD, which effectively binds to Aβ oligomers and increases their clearance is Gammagard Liquid. The monoclonal antibodies bapineuzumab, solanezumab, crenezumab and gantenerumab have also been tested for the same purpose.^25^^,^^28^^,^^29^^,^^30^^,^^31^^,^^32^

Active immunization with synthetic peptides has been tested with the aim of reducing Aβ aggregation caused by the pro-inflammatory signals involved in AD. However, some vaccines that have been tested in relation to AD have presented certain difficulties relating to side effects such as encephalitis and inflammation in the CNS.^25^^,^^28^ One example is Affitope, a patented drug consisting of synthetic peptides with a mechanism of action on AD that so far remains not very well understood.^29^

Many vaccines and drugs relating to monoclonal antibodies have been tested and are protected by patents. These are very effective with regard to active immunization for treating AD. However, the big challenge is to ascertain how safe these interventions are, because some promising forms of immunotherapy have shown serious toxic effects.^26^^,^^29^

### Anti-inflammatory drugs

#### Prednisone, prednisolone, ibuprofen, rofecoxib, naproxen, flavonoids, sulindac sulfide and indomethacin

The inflammation caused by proinflammatory signals that are highlighted by cytokines during the process are characteristic of regions of Aβ plaque mediated by astrocytes and microglia cells. This process promotes continual deposition of Aβ, and therefore some anti-inflammatory drugs have been tested.^21^^,^^25^^,^^26^^,^^33^^,^^34^

It is noteworthy that prednisone and its active metabolite prednisolone are effective in reducing formation of Aβ oligomers *in vitro* , but their effectiveness has not been proven in phase II and III clinical trials. Evidence has emerged from studies on animals that selective inhibitors of cyclooxygenase (ICOX2) are effective, but they are not recommended for chronic treatment.^21^^,^^25^

Ibuprofen, rofecoxib and naproxen have been shown to be able to reduce Aβ levels in animals but not cognitive decline in human trials. However, the possibility that these might help reduce the incidence of the disease rather than its course has recently been investigated.^21^^,^^25^^,^^26^^,^^33^ It is important to highlight natural compounds with anti-inflammatory activity, such as flavonoids. There is evidence from experimental studies on animals that flavonoids prevent cognitive impairment. However, this has been contradicted in some observational studies.^4^

This drug class can be considered to have multiple targets for AD, because some anti-inflammatory drugs have presented activity modulating gamma-secretase, an enzyme involved in formation of NP and NFTs. Ibuprofen, sulindac sulfide and indomethacin were the first non-steroidal anti-inflammatory drugs (NSAIDs) to be reported to decrease Aβ through this mechanism. A Phase III clinical study on r-flurbiprofen in 2009 showed that this agent had good capacity for reducing Aβ, thereby changing the course of AD.^35^

### Inhibitors or modulators of beta and gamma-secretase

#### Pirezenpine, pseudopeptides based on beta-secretase inhibitor, hydroxyethylene, hydroxyethylamine base, carbinamine base, non-peptidomimetics, macrocyclic inhibitors, acylguanidine base, aminoimidazole base, aminohidantoin, aminoquinozoline base, molecules with diversification of non-peptide substrates, semagacestat, avagacestat and malonamide

The preponderant factors in the physiopathology of AD via amyloids involve outflow of Aβ from the brain and formation of insoluble aggregates. The enzymes beta and gamma-secretase are heavily involved in this process. Some drugs that have been studied are able to interfere with the functionality of these enzymes, thereby controlling these pathogenic factors. Although it has been questioned whether enzymes are an important therapeutic target in relation to AD, some molecules that are protected by patents have been studied with regard to such treatments.^29^^,^^36^^,^^37^^,^^38^

Pirezenpine, a muscarinic receptor antagonist for acetylcholine, has shown evidence of capacity for regulating the activity of beta-secretase. In studies conducted in Japan, another molecule called CTS-21166 (which is an analog of pirezenpine) has been shown to decrease formation of Aβ plaque *in vivo* .^39^ Pseudopeptides that inhibit beta-secretase enzymatic activity are noteworthy: replacement of a leucine-alanine statin derivative has led to a variety of potent inhibitors. These include hydroxyethylene, hydroxyethylamine base, carbinamine base; non-peptidomimetics, macrocyclic inhibitors (molecules modified for stability of bioactivity), acylguanidine base, aminoimidazole, aminohidantoin base, aminoquinozoline molecule-based diversifications and non-peptide substrates.^36^

Some compounds such as gamma-secretase inhibitors are currently being studied. Among these are semagacestat, avagacestat and benzodiazepine (under patent), and malonamide. The drug-modulating activity of this enzyme comprises several perspectives because it has lower toxicity than that of inhibitors.^35^

The biggest challenge is to put greater amounts of selective gamma-secretase into the neurons affected by AD.^29^^,^^35^ It is also important to emphasize the challenge of achieving effectiveness with regard to inhibiting or reducing cognitive decline coupled with absence of toxicity, in phase II and III studies. It is possible that structural modifications to these molecules will improve their effectiveness.^36^^,^^37^

### Agents for treating diabetes mellitus

#### Intranasal insulin, rosiglitazone and pioglitazone

Some drugs in this class have been emphasized for treating AD because they have shown evidence of effectiveness in decreasing the accumulation of Aβ oligomers in *in vitro* brain studies. There is also evidence that they decrease the neuronal inflammatory processes that lead to cell death. Intranasal insulin may be an effective way to prevent or treat this disease, since it has significant action in the hippocampus (a region with a large concentration of insulin receptors), thereby improving memory.^40^^,^^41^

Some glitazones have been studied as important drugs for AD, and rosiglitazone and pioglitazone are among these. However, no precise conclusions regarding the evidence for their effectiveness have been reached, and some researchers have condemned them as unpromising drugs.^22^^,^^42^^,^^43^

### Retinoids

#### Tamibarotene

Retinoids have been attributed with great prospects because of the ability of the oligomers to suppress accumulation of Aβ and to decrease some brain inflammation. One example of these drugs is tamibarotene, which has presented promising results. These results showed its effectiveness in stimulating emotional function, through regeneration of cholinergic and glutaminergic nodes. In addition, treatment with this drug has shown improved cognition and orientation with regard to timelines. The expectations for this drug have been strengthened through its good tolerability with prolonged use.^44^

### Statins: lipid-modifying agents

#### Simvastatin and lovastatin

Action by the apolipoprotein E subtype APOE4, which is expressed by the allele APOE Ɛ4, can lead to greater propensity for binding between Aβ oligomers to form NPs. It can also increase the activity of glycogen synthase kinase 3beta (GSK 3beta), thereby causing hyperphosphorylation of tau proteins and formation of NFTs. In some epidemiological studies, statins have shown significant reduction of formation of Aβ plaque in individuals with this protein subtype.^20^^,^^26^

Additionally, one form of action that is already well known for this drug class and which makes it a multitarget drug for AD is its lipid-lowering action. This association is based on scientific evidence linking reduction of systemic cholesterol to neuron preservation in some cases of dementia. There is also evidence of a mechanism for reduction of cholesterol levels in the brain that leads to reduction of NFTs and some pleiotropic effects attributed to anti-inflammatory properties.^20^^,^^45^

Use of statins may be an important adjunct in treating AD. Some physicians have advocated their use as a preventive measure for patients who have a dementia risk profile mediated by cerebral vasculature. However, this class of drugs (such as simvastatin and lovastatin) has great ability to cross the BBB. Therefore, the priority in using these drugs is to promote improvement of circulation loci in the CNS. On the other hand, there is insufficient evidence to recommend their use as a preventive measure or for modifying the course of AD. Furthermore, it needs to be borne in mind that statins have a risky safety profile and there may be hepatic impairment and risk of rhabdomyolysis at doses that would be effective for continuous treatment of AD.^18^^,^^20^

In a meta-analysis on separately evaluated cross-sectional and longitudinal observational studies (total of 19), the cross-sectional studies showed that statins had a protective effect, and it was noted that use of statins was associated with a reduced risk of developing AD and other forms of dementia. However, progress in such studies is still restricted by bias in some of them.^45^

### Nicotinic receptor agonists

#### Nicotine and cotinine

Cotinine is a metabolite of nicotine and it has pharmacological effects similar to those of nicotine. It acts on nicotinic acetylcholine receptors but with lower side effects. Its pharmaco- dynamic properties have been investigated with regard to treating AD because of its neuroprotective capacity over the course of the disease. Cotinine is able to prevent hyperphosphorylation of tau protein denaturation and thus has been shown to reduce neuronal death *in vitro* . Use of this substance has shown evidence of memory improvement, along with a certain degree of safety profile, in phase II clinical trials.^22^^,^^46^

### Polyphenols

#### Curcumin

Some polyphenols have been tested on the basis of the hypothesis that they increase tau protein clearance when they are destructured, as in AD. Thus, their effectiveness has been highlighted in terms of reductions of NFT formation and neuronal death. Curcumin is noteworthy: it is classified as anti-tau, given that it increases production of the anti-inflammatory cytokine IL-4 and reduces tau Aβ levels *in vivo* . It can be highlighted that many molecules in this class of natural products are being studied and some synthesis routes have been patented.^39^

### Phosphodiesterase inhibitors

#### Vinpocetine, rolipram, roflumilast, vardenafil, sildenafil, tadalafil and papaverine

Phosphodiesterase inhibitors have been tested in relation to AD because they act by decreasing gene expression in the cAMP pathway for production of tau and thereby minimize the disintegration process. This leads to a possible reduction in formation of NFTs. These drugs have shown effectiveness in animal testing, in which they have restored cognitive impairment and memory. Among the most effective drugs are vinpocetine, rolipram, roflumilast, vardenafil, sildenafil, tadalafil, papaverine and other molecules under patent.^22^^,^^47^

### NMDA receptor antagonists

#### D-cycloserine and nitromemantine

In the literature, there are studies that have justified increases in hyperphosphorylated tau protein levels and increases in production of βA oligomers through the hypothesis of loss of neuronal homeostasis. This hypothesis is explained by increased glutamatergic activity, i.e. there is an increase in the action of glutamate at NMDA receptors, which leads to excitotoxicity and cell death. Thus, some drugs have been tested in an attempt to modulate NMDA receptors so that they can decrease the formation of amyloid-β plaque and NFTs in AD.^25^^,^^48^^,^^49^

D-cycloserine is an antibiotic capable of modulating the activity of NMDA receptors. It is capable of improving memory and cognitive processes. However, no evidence for its clinical effectiveness has been shown in randomized trials. This means that in studies on AD, this drug has not presented extensive activity. However, it was argued in a recent study that making changes to its molecule would be instrumental for continuing with new tests towards achieving better results.^25^ Nitromemantine is another drug that has shown good results in animals, through high specificity for NMDA receptors, in addition to having fewer side effects.^49^

### Cyclin-dependent kinase modulators: CDK 5

#### Aminothiazole and roscovitine

The subtype of cyclin-dependent kinase known as CDK 5 is an enzyme that has the function of regulating higher neuron life cycles. However, it is also fundamental to the process of hyperphosphorylation of tau proteins. Drugs that show the prospect of capacity for modulation of their activity in order to treat AD now exist. While most of the drugs tested have not shown effectiveness that would justify their use in AD therapy, aminothiazole and roscovitine have shown promise in this regard. These alternatives have shown good efficacy *in vitro* , but they binds to the CDK 5 site without much specificity, thereby leading to side effects that would be serious *in vivo* .^30^

### Antioxidants

#### Vitamin E, selegiline, Ginkgo biloba, resveratrol, vitamin B12, carotenoids, ascorbic acid, catalase, glutathione peroxidase, caffeine, selenium, melatonin, omega-3, silibinin, palmatine, berberine and ubiquinone 

Some studies have shown that cellular oxidative stress is a major aggravating factor in the course of AD. Before the pathophysiological process takes place, some protein, lipid and glicidic oxidation may contribute towards the inflammatory processes and result in emergence or progression of NP and NFTs. Therefore, some compounds with antioxidant activity have been tested with a view to prevention and treatment of AD. These have the capacity to reduce reactive oxygen species (ROS) and interfere with the pathophysiological course of the disease.^18^^,^^34^

One example of this pharmacological subgroup that has been a major subject of study is the vitamin E substance known as α-tocopherol. Clinical trials have shown the ability of this compound to reduce the chance of developing AD over a four-year follow-up period by about 2.5 to 4.0-fold. Although there is sufficient evidence to show that this substance can influence the course of AD, there are few studies that have measured its great potential as an adjunct to treatment.^25^^,^^26^^,^^34^^,^^48^

Another drug that has shown promise in studies on AD is selegiline. Eight clinical trials on this drug involving twelve evaluators have shown evidence of improvement in mood and behavior. Additionally, significant benefit for memory has been shown in a meta-analysis. Although the results relating to this drug have been good, there is not enough evidence to include it in treatments for AD.^25^^,^^26^^,^^34^

*Ginkgo biloba* produces a substance belonging to the class of cyclic diterpenes that is a nootropic drug that has significant effects as a multitargeted drug for treating AD. It is effective in this treatment because of its ability to increase blood flow in the microcirculation and its antioxidant properties, which prevent reduction of synapses and increase the production of neurotrophic factors relating to neuronal apoptosis in AD. Therefore, it is capable of causing behavioral improvements regarding the damage induced by Aβ and positive effects on perception and memory. This substance has significant side effects that have been investigated. These include bleeding when anticoagulation therapy or drugs likely to cause bleeding are used concomitantly. However, no side effects have been seen when it is used alone.^25^^,^^27^^,^^34^^,^^50^

Other drugs with antioxidant action in relation to AD include resveratrol (a compound from grapes), vitamin B12, carotenoids, ascorbic acid (vitamin C), catalase, glutathione peroxidase, caffeine, selenium, melatonin, omega 3, silibinin flavonoids, palmatine alkaloids, berberine and ubiquinone terpenoids.^26^^,^^34^^,^^51^

### Phospholipase A2 inhibitors

#### Methyl arachidonoyl fluorophosphate, arachidonoyl trifluoromethyl ketone, Ginkgo biloba, curcumin and epigallocatechin gallate

Some studies have shown that abnormal phospholipase A2 (PLPA2) activity implies the existence of some neurodegenerative pathogens, including AD. Although this association is currently not well understood, it is believed that prolonged neuron exposure to Aβ may gradually give rise to mitochondrial dysfunction and stimulate activation of PLPA2 through increasing ROS and excitotoxicity.^25^^,^^34^

Two compounds that have shown effective results *in vitro* with regard to this mechanism are methyl arachidonoyl fluorophosphate and arachidonoyl trifluoromethyl ketone. These have shown good ability for modulating the properties of neuronal membranes and increasing protection against the neuronal impairment caused by Aβ plaque. Studies have shown a correlation between neuroexcitatory PLPA2 glutamate receptors and NMDA capability in relation to stimulating production of ROS through NADPH oxidase. *Ginkgo biloba*, curcumin and epigallocatechin gallate (polyphenols) are substances capable of protecting against the pathological course of AD through this mechanism.^34^^,^^52^

### Nootropic drugs without an elucidated mechanism of action for Alzheimer's disease

#### Nicergoline, piracetam and aniracetam

This pharmacological class covers drugs that enhance cognitive performance and, as such, are targets for evaluation with a view to treating psychobehavioral symptoms relating to dementia. In addition, they have the significance of being drugs that have passed through phase IV clinical studies, have been on the market for a while and have some degree of safety profile.^25^^,^^53^

Nicergoline, a derivative of ergot, has positive effects on behavior and cognition in AD. Piracetam is a derivative of gamma-aminobutyric acid (GABA) that binds to the neuronal membrane, thus promoting formation of active phospholipid complexes. This increases lamellar restoration, thereby improving perception and memory. *In vivo* studies have shown evidence that this improves memory retention. However, some reviews on this drug have not shown any evidence for its applicability in treatments for AD.^25^

Aniracetam is a drug that has shown good tolerability in relation to treating AD. Experimental data have suggested that this medication presents interactions with multiple neurotransmitter systems. In monotherapy, it has shown clinical results that are more effective than those from conjugated cognition therapy and ICHE monotherapy for starting the treatment. Over the long term, it has shown improved functional capacity in relation to depression and improvement in the physiopathology of dementia only when combined with ICHE. However, it has been found to present some adverse events that counter its supposed effectiveness and which do not corroborate its indication for treating AD.^53^

### Agents for hormone therapy: hormones

#### Estrogen, conjugated estrogens and medroxyprogesterone

It has been hypothesized that hormone levels in the postmenopausal period provide physiological mechanisms that trigger AD. Thus, some research has been developed in order to investigate this idea. Based on the notion that decreased estrogen levels combine with an increased chance of developing AD, studies have attempted to evaluate therapy using raloxifene. However, this issue remains controversial. A study on postmenopausal women on estrogen replacement therapy and without this therapy revealed that in the first group, 4% of the women had AD, and that in the second group, 10% had AD. However, reviews and meta-analyses indexed in the Cochrane Library have not found any evidence to show that estrogen replacement therapy is effective for preventing and treating AD.^25^^,^^54^

Some studies have shown that therapy consisting of a combination of estrogen and progesterone before the menopause was able to reduce the risk of AD, although, this association may increase the risk of developing AD after the age of 65 years. It is noteworthy that conjugated estrogen therapy in association with medroxyprogesterone showed a clear clinical response in terms of memory and aphasia among both young women and postmenopausal women.^54^

### Chelating agents

#### Desferrioxamine and copper and zinc chelating agents

Chelating agents exhibit efficacy through removal of excess ferric ions and other ions such as copper, aluminum and zinc, which may be related to neurotoxicity through formation of free radicals that bind to Aβ peptides. One drug that has been studied for this purpose in relation to treating AD is desferrioxamine. However, it has shown retinoid toxicity.^35^ Copper and zinc chelating agents have also shown the ability to mediate aggregation of Aβ peptides. However, many of these drugs present concerns regarding their safety profile, given that they are responsible for causing optic neuritis.^18^^,^^25^^,^^26^

### Antihypertensive agents: calcium channel blockers

#### Isradipine

Antihypertensive drugs that are calcium channel blockers reduce the influx of Ca^2+^ ions into Aβ channels, thereby minimizing and reducing the neurotoxicity of Aβ formation *in vitro* . Isradipine has been tested in clinical trials as monotherapy and has already shown positive effects in relation to treating AD based on this mechanism.^55^

Six broad experimental studies that were double-blind, randomized and placebo-controlled investigated about 50 antihypertensive medications for treating dementia and cognitive decline. These studies suggest that treatment with antihypertensive drugs may play an important role in preventing dementia, thereby producing notable cognitive improvement. The important effect of calcium channel blockers on this line of treatment was emphasized.^56^

The effectiveness of this alternative drug treatment for AD becomes more pronounced when antihypertensive agents are used in association with other classes of drugs. A combination of calcium channel blocker, angiotensin-converting enzyme (ACE) inhibitor and diuretic was found to have reduced the incidence of dementia in AD cases after two and four years of follow-up by 50% and 55% respectively.^56^ A combination consisting only of ACE inhibitor and diuretic reduced the occurrence of dementia by 31%. Thus, this suggested that treatment with calcium channel blockers helped in preventing dementia.

Nonetheless, the evidence from studies remains insufficient to be able to state that this class of medication should be used for treating AD. Therefore, it is essential to conduct clinical studies with greater robustness regarding use of antihypertensive treatment, since the evidence suggests that the effectiveness of this class of drugs with regard to prevention of AD relates to specific cases such as dementia of microcirculatory origin.^26^^,^^56^

## CONCLUSION

From the studies analyzed, it could be seen that there is a current trend among researchers towards separating out the dementia phase from the preclinical phase at which AD usually usually starts. This trend has influenced the search for new drugs. In addition, we can conclude from the results of this study that there are no promising drugs capable of providing effectiveness and safety. In this context, immunomodulators are more likely to become drugs capable of influencing the course of AD, because the studies selected showed better quality and the results were promising. However, the toxicity of these drugs for treating AD constitutes a major obstacle. This was also observed in relation to new drugs that can interfere with alternative targets, such as inhibitors or modulators of gamma or beta-secretase, phosphodiesterase inhibitors, cyclin-dependent kinase modulators (CDK 5) and phospholipase A2 inhibitors. However, the evidence relating to these drugs is weak, as shown by the quality of the articles accessed in this review. No robust studies have yet been conducted, and this is due to the high toxicity of these drugs in relation to treating AD.

One alternative for conducting successful searches relating to the safety and effectiveness profiles of drugs for treating AD would be to use molecular modeling investigations or combination therapies such as statins and antihypertensive drugs. However, even though the articles accessed in relation to these therapeutic groups were of good quality, these are drugs without any clear mechanism of action. These drugs would probably be effective for some types of AD, such as disease of vascular origin in the preclinical phase, and they might not interrupt the course of the disease.
